# Antibodies Targeting Closely Adjacent or Minimally Overlapping Epitopes Can Displace One Another

**DOI:** 10.1371/journal.pone.0169535

**Published:** 2017-01-06

**Authors:** Yasmina Noubia Abdiche, Andy Yik Yeung, Irene Ni, Donna Stone, Adam Miles, Winse Morishige, Andrea Rossi, Pavel Strop

**Affiliations:** 1 Department of Protein Engineering, Rinat, Oncology Research and Development, Pfizer Inc., South San Francisco, California, United States of America; 2 Wasatch Microfluidics, Salt Lake City, Utah, United States of America; 3 Department of Protein Engineering, Bristol-Myers Squibb, Redwood City, California, United States of America; 4 Compugen USA Inc., South San Francisco, California, United States of America; New York State Department of Health, UNITED STATES

## Abstract

Here we describe how real-time label-free biosensors can be used to identify antibodies that compete for closely adjacent or minimally overlapping epitopes on their specific antigen via a mechanism of antibody displacement. By kinetically perturbing one another’s binding towards their antigen via the formation of a transient trimolecular complex, antibodies can displace one another in a fully reversible and dose-dependent manner. Displacements can be readily identified when epitope binning assays are performed in a classical sandwich assay format whereby a solution antibody (analyte) is tested for binding to its antigen that is first captured via an immobilized antibody (ligand) because an inverted sandwiching response is observed when an analyte displaces a ligand, signifying the antigen’s unusually rapid dissociation from its ligand. In addition to classifying antibodies within a panel in terms of their ability to block or sandwich pair with one another, displacement provides a hybrid mechanism of competition. Using high-throughput epitope binning studies we demonstrate that displacements can be observed on any target, if the antibody panel contains appropriate epitope diversity. Unidirectional displacements occurring between disparate-affinity antibodies can generate apparent asymmetries in a cross-blocking experiment, confounding their interpretation. However, examining competition across a wide enough concentration range will often reveal that these displacements are reversible. Displacement provides a gentle and efficient way of eluting antigen from an otherwise high affinity binding partner which can be leveraged in designing reagents or therapeutic antibodies with unique properties.

## Introduction

**Fig 1:**
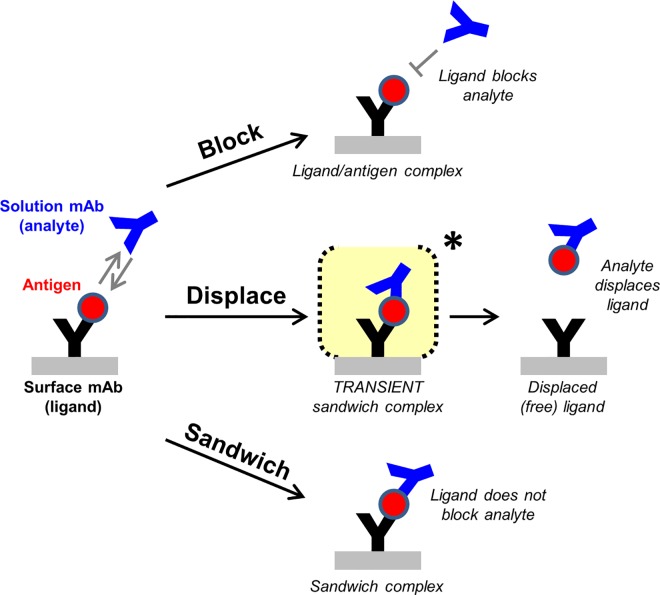
Schematic of an epitope binning assay conducted in a classical sandwich format. One of three outcomes is typically observed when two mAbs are competed against one another for binding to their specific antigen; (A) block, (B) displace, or (C) sandwich pair.

High-throughput epitope binning experiments on real-time label-free biosensors are commonly used in early stage discovery of therapeutic monoclonal antibodies (mAbs) to sort large panels of mAbs into epitope families or “bins” based upon their ability to block one another’s binding to their specific antigen in a pairwise and combinatorial fashion. A bin is a relative term defining a mAb’s “blocking fingerprint” relative to others in the test set [1]. Since bin members are likely to share similar functional characteristics, these analyses inform the selection of a subset of mAbs, representative of the panel’s epitope diversity, for further testing in biological assays to identify the bins that target epitopes of interest, such as those that block the antigen’s natural function [2]. In a “classical sandwich” epitope binning assay format [1], a solution mAb (or analyte) is tested for binding to its specific antigen that is first captured via an immobilized mAb (or ligand). One of two outcomes is typically observed; a) the mAb analyte is blocked, as judged by its lack of binding response, suggesting that the analyte and ligand compete for overlapping epitopes, or b) the mAb analyte is not blocked, as judged by its significant binding response indicating the formation of a trimolecular or “sandwich” complex due to the analyte and ligand co-existing on their antigen at non-competing and non-overlapping epitopes. However, some mAb pairs cannot be classified unambiguously as blocked or not blocked because mAbs can also compete with one another by kinetically altering one another’s binding to their specific antigen via the formation of a transient trimolecular complex, which then rapidly collapses by retaining one mAb and displacing the other. This novel third phenomenon of mAb displacement may be considered a hybrid between a block and a sandwich, resulting in the antigen exchanging binding partners (Fig 1). The time-course of a displacement can be readily monitored when mAb competition is examined in a classical sandwich epitope binning assay format using real-time and label-free biosensors.

Here, we explore the molecular mechanism of mAb displacement as observed in the context of epitope binning experiments, to better understand the epitope requirements for this phenomenon to occur. By characterizing four panels of mAbs targeting unrelated protein antigens—namely human proprotein convertase subtilisin/kexin type 9 (PCSK9), human progranulin (PGRN), human epidermal growth factor receptor (EGFR), and hen egg white lysozyme (HEL)—we find that displacements appear to occur on any antigen, if the mAb panel contains appropriate epitope diversity. To rationalize our empiric cross-blocking assignments of block, displace, or sandwich (Fig 1), we use structural data for a subset of literature mAbs binding EGFR or HEL and find that mAbs with closely adjacent or minimally overlapping epitopes can displace one another. Antibody displacement can be driven to completion by increasing the concentration of the mAb analyte, allowing a lower affinity antibody to displace a higher affinity antibody. Displacement therefore offers a molecular mechanism by which mAbs can compete with one another in a potent manner that is not limited by affinity, which can be leveraged in drug discovery to identify binders with novel binding modes.

## Materials and Methods

### Antibodies

Anti-PCSK9 mAbs were generated from an in-house in vitro library and expressed recombinantly as human IgG molecules, except mAb C34, which was produced in collaboration with Crystal Bioscience from the immunization of chickens and expressed recombinantly as a single-chain Fv fused to a human IgG1 Fc fragment (giving a final homodimer molecular weight of 100 kDa). Anti-PGRN mAbs were generated in-house from the immunization of mice using standard hybridoma methods and purified by protein A chromatography, except the chicken antibody mAb C21, which was produced by Crystal Bioscience, as above. Anti-EGFR mAbs 54D7 and 17D7 were produced in-house from the immunization of mice using standard hybridoma methods. Cetuximab, necitumumab (IMC-11F8), duligotuzumab (DL11), and matuzumab, corresponding to Protein Data Bank Identification (PDB ID) codes 1YY9, 3B2U, 3P0Y, and 3C09, respectively [3–6] were produced as Fab fragments by co-transfection of heavy and light chain vectors using an in-house Expi293 transient expression system. Histidine-tagged Fab fragments were purified via standard immobilized metal ion affinity chromatography (IMAC) using Ni Sepharose^®^ Excel (GE Healthcare, catalog number 17-3712-02). Recombinant purified anti-HEL mAbs cAb-Lys2, F10.6.6, cAb-Lys3, and D11.15, corresponding to PDB ID codes 1RJC, 1P2C, 1JTP, and 1JHL, respectively [7–10], were provided as full-length human IgG1 molecules as part of a collaboration with Adimab LCC [11]. Note that cAb-Lys2 is a camelid single domain antibody (VHH), here fused to the Fc portion of a human IgG1 (hinge plus CH2 and CH3 domains), giving a homodimer molecular weight of 80 kDa.

### Biosensor Methods

#### General methods

Biosensor experiments were performed on various surface plasmon resonance (SPR) platforms, including ProteOn XPR36 (BioRad, Inc), Biacore 2000 and Biacore T200 (GE Healthcare Life Sciences), and MX96 SPR imager (Ibis technologies B.V.) and on biolayer interferometry platforms, Octet Red384 and Octet HTX (ForteBio, Pall Inc), as indicated. All experiments were conducted at 25°C and in a running buffer of PBS + 0.01% Tween-20 (ProteOn and MX96), PBS + 0.05% Tween-20 (Biacore), or PBS + 0.05% Tween-20 + 5 g/l BSA (Octet). Antibodies were lightly biotinylated using a 3:1 molar ratio of EZ-Link™ Sulfo-NHS-LC-Biotin (Thermo Fisher Scientific, catalog number 21335) to protein to enable their capture via streptavidin or neutravidin surfaces. Anti-PCSK9 surfaces were regenerated with a 2:1 v/v mixture of Pierce IgG Elution Buffer pH 2.8 (Thermo Fisher Scientific, catalog number 21004):4 M NaCl, whereas all other surfaces were regenerated with 75 mM phosphoric acid. Specific experimental details are provided below.

#### ProteOn one-shot kinetic analysis

The binding kinetics of monovalent antigens (PCSK9, PGRN, and EGFR) to their respective immobilized mAbs were determined using a one-shot kinetic method [12] on a ProteOn XPR36 biosensor equipped with GLC or NLC (neutravidin-coated) sensor chips. Antibodies were amine-coupled onto GLC chips or biotinylated and captured onto NLC chips. Analytes were injected for 3 min at appropriate concentration ranges (typically as a 3fold or 5fold dilution series) in duplicate binding cycles allowing up to 3 h dissociation time, depending on the experiment. Surfaces were regenerated after each binding cycle. Binding data were double-referenced [13] and analyzed globally using a Langmuir model with mass transport in the ProteOn Manager software. For very stable interactions that showed < 5% decay in their binding responses during the allowed dissociation phase, a limit was placed on their reported k_d_ value, per the 5% rule [14].

#### Biacore kinetic analysis

A panel of anti-PCSK9 mAbs was kinetically screened on a Biacore T200 equipped with a CM4 sensor chip. To prepare the capture surfaces for these experiments, goat anti-human IgG Fc polyclonal antibody (Southern Biotech, catalog number 2014–01) was amine-coupled onto all four flow cells using a standard procedure, except that surfaces were blocked with 0.1 M ethylenediamine in 0.2 M sodium borate buffer pH 8.5 (instead of using 1.0 M ethanolamine-HCl pH 8.5), yielding final coupled levels of approximately 3,800 RU per flow cell. Reaction surfaces were prepared by capturing anti-PCSK9 mAbs (as ligands) at approximately 4 μg/ml onto individual flow cells (2, 3 or 4) for 30 sec, giving capture levels varying from 29 RU to 195 RU. Flow cell 1 was left blank to serve as a reference surface. Each mAb was injected in three cycles, varying the analyte concentration used per cycle (0, 5, or 100 nM PCSK9). Following a 15-min dissociation phase, the capture surfaces were regenerated. Binding data were double-referenced and analyzed globally using a Langmuir model with mass transport in the Biaevaluation T200 software.

#### Array-based epitope binning assays

High-throughput epitope binning experiments on PCSK9 were performed in a classical sandwich assay format using an MX96 SPR imager in a 96-ligand array configuration, as described previously [15], but using more sophisticated software for the data analysis (ECTO module 2.0 version 33, Wasatch Microfluidics) that help to identify (and discard) non-ideal or problematic behaviors from ligands (coupled mAbs) and analytes (solution mAbs). Representative sensorgrams obtained for multiple binning cycles on a single spot are shown as overlay plots (S2, S3 and S6 Figs). It is noteworthy that the sharp drop in signal between the antigen capture step and the antibody analyte step is an artifact produced by the MX96 SPR imager because it lacks active valving near the flow cell and therefore cannot perform a true back-to-back injection of two samples (also known as a “coinject”). The queuing of the antigen injection and the secondary antibody causes a slight delay, with buffer on the surface as the secondary antibody plug is transported. The MX96 is not collecting data during the queueing step, hence the downward drop to connect (or “stitch together”) the two measured points. The curated binning results are displayed in terms of a heat map, with analytes as columns and ligands as rows, and cells colored red or green to indicate analyte/ligand pairs that show blocked or sandwiching responses, respectively, according to user-defined threshold settings. Analyte/ligand pairs that yield responses in-between these thresholds are colored yellow to indicate their intermediate or unassigned blocking status. The clustering of mAbs per their blocking behaviors is also graphed as a network blocking plot, defining “bins” (most granular assignment) or “communities” (less granular assignment), enclosed by the envelopes. The granularity of the analysis depends on the cut-off used for comparison. In these plots, chords represent the blocking relationships; a solid chord connects two mAbs that block or displace one another in both orientations and a dotted chord connects two mAbs that block or displace one another in only one orientation.

#### Waterfall competition analyses

A Biacore 2000 equipped with SA (streptavidin-coated) sensor chip was used for low-throughput epitope binning assays conducted in a classical sandwich assay format with titrating levels of the mAb analyte, herein referred to as “waterfall competition” experiments. Biotinylated mAbs were captured at low levels (< 500 RU) onto flow cells 2, 3, or 4, to provide the reaction surfaces, leaving flow cell 1 blank to serve as a reference surface. Waterfall competition was performed using a coinject strategy, whereby the antigen was injected at a constant concentration (typically 40 nM) and followed immediately by an injection of a mAb analyte as a 3fold dilution series, varying the mAb analyte’s concentration cycle-to-cycle. A top mAb analyte concentration of 300 nM binding sites was used for the PCSK9 experiments and 3000 nM was used for the EGFR experiments. In the example where 100 nM anti-PCSK9 mAb 69 analyte fully displaced mAb C34 ligand, no regeneration was used. Similar waterfall competition experiments were performed on the ProteOn for PGRN and the Octet Red384 for EGFR and HEL.

#### Premix competition

A ProteOn XPR36 was used to perform competition experiments of anti-PCSK9 mAb 69 and mAb C34 in a premix assay format, allowing each mAb the role of analyte (premixed with PCSK9 in solution) and ligand (on chip). The same surfaces, running buffer, and regeneration conditions were used as those for one-shot kinetics. The samples were prepared by mixing 8 nM PCSK9 with 0, 4, 8, 16, 32, or 64 nM mAb binding sites and allowing the mixtures to equilibrate before injecting them over the surfaces for 3 min, allowing a 3-min dissociation time. Data were double-referenced, as above.

Premix competition of anti-PGRN mAbs 14C7, 28H6, and C21 was analyzed on an Octet Red384 equipped with streptavidin sensors, onto which biotinylated mAbs were captured. Premixed samples were prepared by incubating 40 nM PGRN with 0, 1.4, 4.1, 12.3, 37, 111, 333, or 1000 nM mAb binding sites and allowing the mixtures to equilibrate before exposing them to the mAb-coated sensors for 15 min. Every analyte/ligand pairwise permutation was addressed. Octet data were processed in the control software by simply adjusting the baseline to zero at the start of the binding step.

#### KinExA solution affinity determination

Serum experiments were performed as described previously [15, 16] using a Dylight-labeled anti-PGRN mAb 27D5 (M14) as secondary detection, chosen because its epitope does not overlap with that of mAb 14C7 (M4) or mAb 28H6 (M27).

### Library design, protein expression and screening

Accessible surface area was computed in MODELLER using the WRITE_DATA function at the residue level (PDB file 1NQL). Residue positions, except for cysteines, prolines and glycines, were sorted based on percentage of side-chain solvent accessibility. The top 192 residue positions with highest solvent accessibility were selected for mutagenesis. Each position was mutated to alanine and glutamate, except for alanines (mutated to tyrosine and glutamate) and glutamates (mutated to alanine and threonine). Mutants (with Histidine tag) were generated by standard molecular biology techniques. Protein expression was performed by standard transient transfection in Expi293 cells in 96 deep well plates. A total volume of 0.7 ml per well was incubated for four days and shaken at 1400 rpm in 95% humidity environment.A 96-channel mode Octet HTX equipped with streptavidin sensors was used to screen the expressed EGFR mutants in crude supernatant form for binding to mouse IgGs 17D7, 54D7 and cetuximab Fab. His-tagged EGFR mutants were captured via biotinylated anti-His mAb (R&D systems, catalog number BAM050) or via a biotinylated high-affinity anti-EGFR mAb, murine 19F4 (prepared in-house), that binds a different EGFR domain.

#### Structural epitope determination

The epitope residues for the four literature anti-EGFR Fabs (cetuximab, necitumumab, duligotuzumab, and matuzumab) on the EGFR extracellular domain (ECD) were identified by calculating the difference in accessible surface area between the complex of EGFR-ECD with a given mAb Fab fragment and EGFR-ECD structure alone. EGFR residues that show buried surface area upon complex formation with the four Fabs were defined as being part of the structural epitope. The solvent-accessible surface of a protein was defined as the locus of the center of a probe sphere (representing a solvent molecule of 1.4-Å radius) as it rolls over the Van der Waals surface of the protein. The solvent-accessible surface area was calculated by generating surface points on an extended sphere about each atom (at a distance from the atom center equal to the sum of the atom and probe radii), and eliminating those that lied within equivalent spheres associated with neighboring atoms as implemented in the program AREAIMOL (http://www.ccp4.ac.uk/newsletters/newsletter38/03_surfarea.html).

## Results

### Antibody displacement is reversible, dose-dependent and can be used to elute antigens from high affinity binding partners at neutral pH

**Fig 2:**
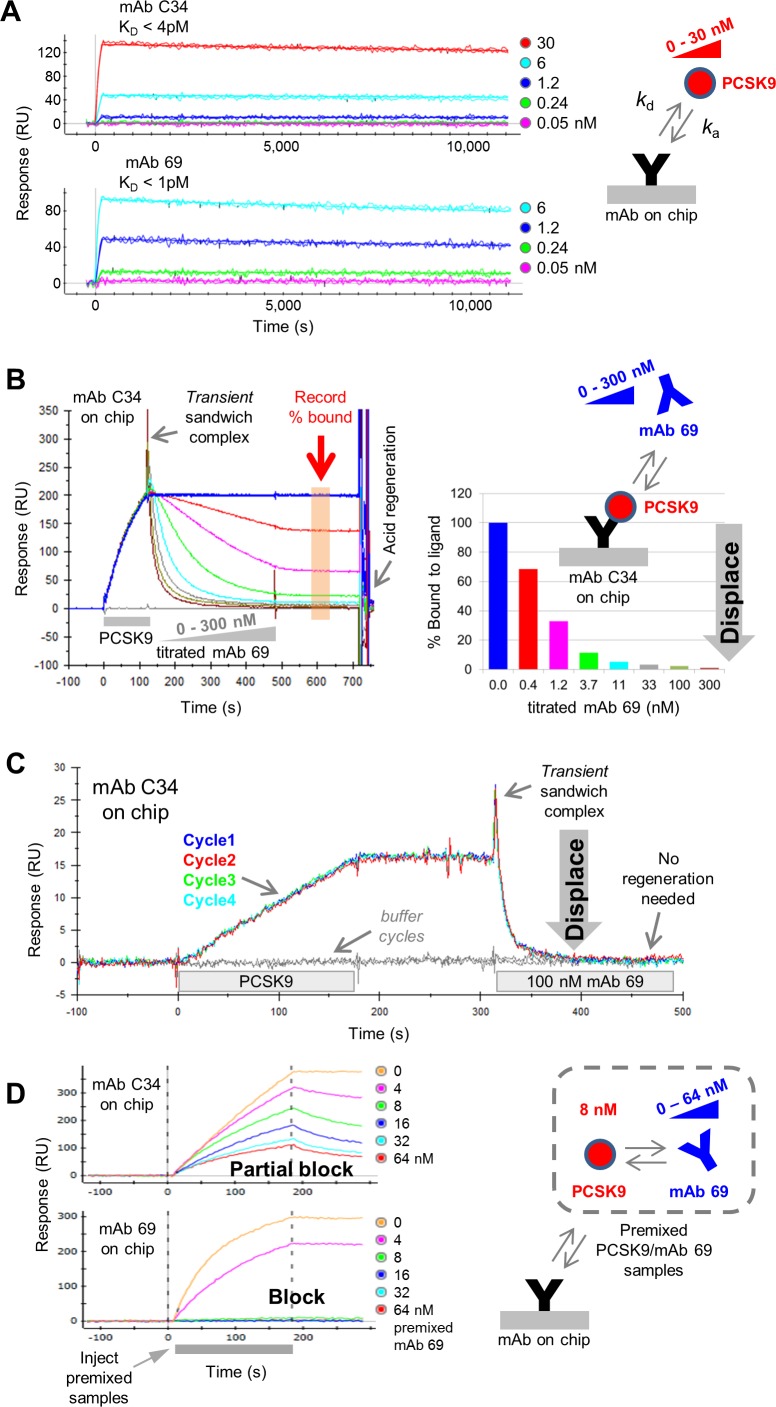
Anti-PCSK9 mAbs 69 and C34 potently displace one another. (A) One-shot kinetic analysis of PCSK9 binding as analyte to C34 or 69 ligands (see Table 1). (B) Biacore 2000 data showing a waterfall competition plot in which mAb 69 analyte dose-dependently displaces mAb C34 ligand, with curves colored by analyte concentration (in binding sites). The binding responses recorded at the end of the analyte injection are graphed in the histoplot and expressed as a percent bound. (C) Biacore 2000 data showing that 100 nM mAb 69 analyte fully displaces mAb C34 ligand reproducibly without the need for any regeneration. (D) Premixing 8 nM PCSK9 with titrating levels of mAb 69 only partially blocks binding to mAb C34 ligand but fully blocks binding to mAb 69 ligand (ProteOn data).

To investigate the mechanism of mAb displacement, we first focus on two mAbs that bind with high specificity and high affinity towards PCSK9, namely mAbs C34 and 69, showing apparent K_D_ values in the single digit picomolar range when analyzed by SPR biosensors (Fig 2A). In each case, PCSK9 bound very stably, with barely any decay of the PCSK9/mAb complex observed during the allowed dissociation time of 3 h. When the competition of mAbs C34 and 69 is tested using a classical sandwich assay format, mAb 69 analyte dose-dependently displaces mAb C34 ligand (Fig 2B); we term this type of analysis, “waterfall competition”. This example shows that a transient analyte/PCSK9/ligand trimolecular complex is observed as a sharp peak during the initial sandwiching step. This is followed by its rapid decay as shown by the set of inverted sandwich response curves, signifying the progressive loss of mass from the mAb C34 ligand with titrating levels of mAb 69 analyte. The rapid dissociation of the PCSK9/C34 complex upon its transient sandwiching with mAb 69 analyte is rather remarkable given that the PCSK9/C34 complex would otherwise dissociate extremely slowly (Fig 2A). In the waterfall competition experiment, 11 nM mAb 69 analyte binding sites appeared to displace 95% of PCSK9 from mAb C34 ligand, as shown by responses falling to near-baseline levels, representing mostly naked (or unbound) mAb C34 ligand (Fig 2B). A similar result is observed upon reversing the assay orientation, namely mAb C34 analyte displaces mAb 69 ligand, but in this orientation the transient sandwich complex is barely detected (data not shown). When mAb 69 analyte is injected at 100 nM, it fully displaces mAb C34 ligand within a few seconds without the need for any regeneration of the surface (Fig 2C). Furthermore, the displaced mAb C34 ligand retains its full PCSK9-binding activity as judged by the highly reproducible sensorgrams obtained for quadruplicate binding cycles. Thus, mAb displacement provides a gentle, yet effective method for rapidly dissociating very stable interactions, which could be applied to eluting proteins from affinity columns.

Competition between mAb C34 and mAb 69 was also examined in a premix assay format.^1^ In this experiment, mAb 69 is titrated into a constant concentration of PCSK9 and these mixtures are allowed to equilibrate before injecting them over mAb C34 or mAb 69 ligands (Fig 2D). In these premixed samples, PCSK9’s concentration is held constant at 8 nM, chosen to be several orders of magnitude above (in this case, 1000fold above) the K_D_ of its interaction with its solution partner (mAb 69) to promote titration under stoichiometrically-controlled, rather than K_D_-controlled, conditions [17]. Injecting these PCSK9/mAb 69 premixed samples over mAb C34 ligand gave a partial block, inconsistent with an exact titration of binding sites, whereas injecting the same samples over mAb 69 ligand gave a full block, consistent with an exact titration of binding sites. Taken together, these observations suggest that mAb 69 ligand detects only “free” PCSK9 in the PCSK9/mAb 69 premixed samples, while mAb C34 ligand detects both, “free” and “mAb 69-bound” PCSK9 consistent with the detection of a short-lived sandwich species (C34/PCSK9/69). A similar result was observed when the assay orientation was reversed, namely premixing PCSK9 with titrating levels of mAb C34 achieved a full self-block on mAb C34 ligand but a partial cross-block on mAb 69 ligand (see S1 Fig).

### The results from high-throughput epitope binning experiments imply that antibodies can displace others with similar blocking profiles

**Fig 3:**
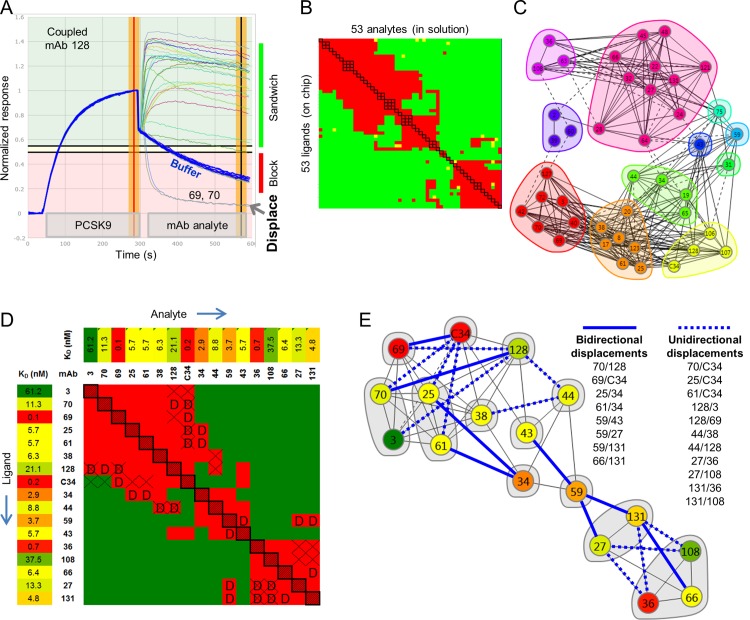
SPR imaging data for an epitope binning analysis on a panel of anti-PCSK9 mAbs. (A) Example of binning in a classical sandwich assay format on a single spot of a 96-ligand array, coupled with mAb 128 (ligand), showing PCSK9’s potent displacement by mAb 69 and mAb 70 analytes. (B) 53 x 53 (analyte x ligand) heat map for a comprehensive pairwise cross-blocking analysis of 43 unique clones (of which 3 were tested in triplicate, 4 were tested in duplicate, and the remaining 36 were tested once). The red, green, and yellow cells represent analyte/ligand pairs that blocked (or displaced), sandwiched, or gave an intermediate response, respectively. (C) Community network plot, colored by community. (D) Distilled heat map including only the subset of mAbs that showed obvious displacements of at least one other mAb (marked “D”), along with their PCSK9-binding affinities (Biacore T200 data). Asymmetries representing a unidirectional block or displacement are marked with “X”. (E) Network blocking plot for the distilled heat map in panel D, colored by affinity. Blue solid chords represent bidirectional displacements and blue dotted chords represent unidirectional displacements, as listed.

Next, we expand the example above to a panel of 43 anti-PCSK9 mAbs that was epitope binned in a classical sandwich assay format using an array-based SPR imager, with every pairwise permutation addressed by testing each mAb in the role of analyte and ligand. An example of the data obtained for multiple rounds of binning cycles on a single spot, in this case coupled with mAb 128, is shown in Fig 3A. Many analytes show clear binding responses to the PCSK9/mAb 128 complex and so are classified as “sandwiching” (shown by curves that fall into the green background), while others show negligible binding, like the buffer responses (blue curves), so are classified as “blocked” (shown by curves that fall into the red background). However, two analytes, mAb 69 and mAb 70, show a third behavior, as evidenced by their inverted sandwich responses that fall below the buffer baseline curves, indicating their rapid removal of mass (PCSK9) from the surface, by their displacing mAb 128 ligand. Due to software limitation in handling these data sets, displaced ligands were classified along with blocked ones in constructing the heat map summarizing the binning outcomes (Fig 3B). This is because the analysis was limited to a binary classification of “block” or “sandwich” as dictated by user-defined threshold settings, allowing an analyte’s normalized response to be partitioned as follows (see Methods); (a) <0.2 = block (red cell in the heat map), (b) 0.2–0.25 = intermediate/ambiguous (yellow cell in the heat map), or (c) >0.25 = sandwich (green cell in the heat map). Since displacements often manifest as inverted (or negative) sandwich responses that fall below the responses of the buffer blanks, which are set to zero for establishing the “blocked” threshold, they are therefore classified along with blocked responses. The results of the heat map are alternately graphed as a network blocking plot, highlighting the epitope communities, or bin clusters, and their interconnectivities (Fig 3C). In this type of plot, mAbs are represented as “nodes” that are colored and grouped per their similarities in blocking behavior, with the blocking relationships represented by chords. Solid chords connect two mAbs that block or displace one another in both assay orientations (e.g., the mAb pair 36/108, in the purple cluster), whereas dotted chords connect two mAbs that appear to block or displace in only one orientation (e.g., mAb 63 in the purple cluster appears to block mAb 24 in the pink cluster only when mAb 63 is the ligand–see S2 Fig). Two mAbs that do not block one another are not connected by a chord, e.g., mAb 108 in the purple cluster sandwich pairs with mAb C34 in the yellow cluster. These epitope communities can change depending on the user-defined cut-off used for comparison.

To highlight the frequency of displacements observed within this panel of mAbs and the complexity this introduces into their blocking assignments, we now focus on a subset of 17 mAbs that showed displacement of at least one other mAb (Fig 3D). The ability of mAbs to displace one another appears reversible, as inferred by the general symmetry of the displacements (marked “D”) within the heat map. Upon visual inspection of the heat map, it appears that displacements cluster at the interface between blocking pairs (red cells) and sandwiching pairs (green cells), which can sometimes result in an ambiguous blocking assignment for mAb pairs that straddle this interface. This is because, when displacements are considered as an additional metric for characterizing cross-blocking outputs, numerous cases of asymmetries (marked “X”) appear, whereby an analyte/ligand pair yields a displacement only in one assay orientation. For example, mAb 69 analyte appears to displace mAb 128 ligand, yet in the reverse orientation, mAb 69 ligand appears to block mAb 128 analyte. In another example, mAb C34 analyte displaces mAb 70 ligand, yet mAb 70 analyte appears to sandwich pair with mAb C34 ligand. In both cases, the apparent “unidirectional” displacement occurs between mAbs with disparate affinities, with the higher affinity mAb analyte showing displacement; the relative SPR-determined affinities of this panel are reported in the heat map and colored using a gradient (Fig 3D). The complexity in blocking behaviors resulting from kinetic perturbations between two transiently sandwiching mAbs that are displacing one another is captured in the network blocking plot (Fig 3E), which is colored by affinity to highlight that unidirectional displacements often arise between affinity-mismatched mAbs. A more detailed analysis of mAb C34’s order-dependent blocking behavior towards various mAbs is shown in S3 Fig.

### Two mAbs with 1000fold different affinities but similar kinetic association rate constants are similarly potent at displacing other mAbs

**Fig 4:**
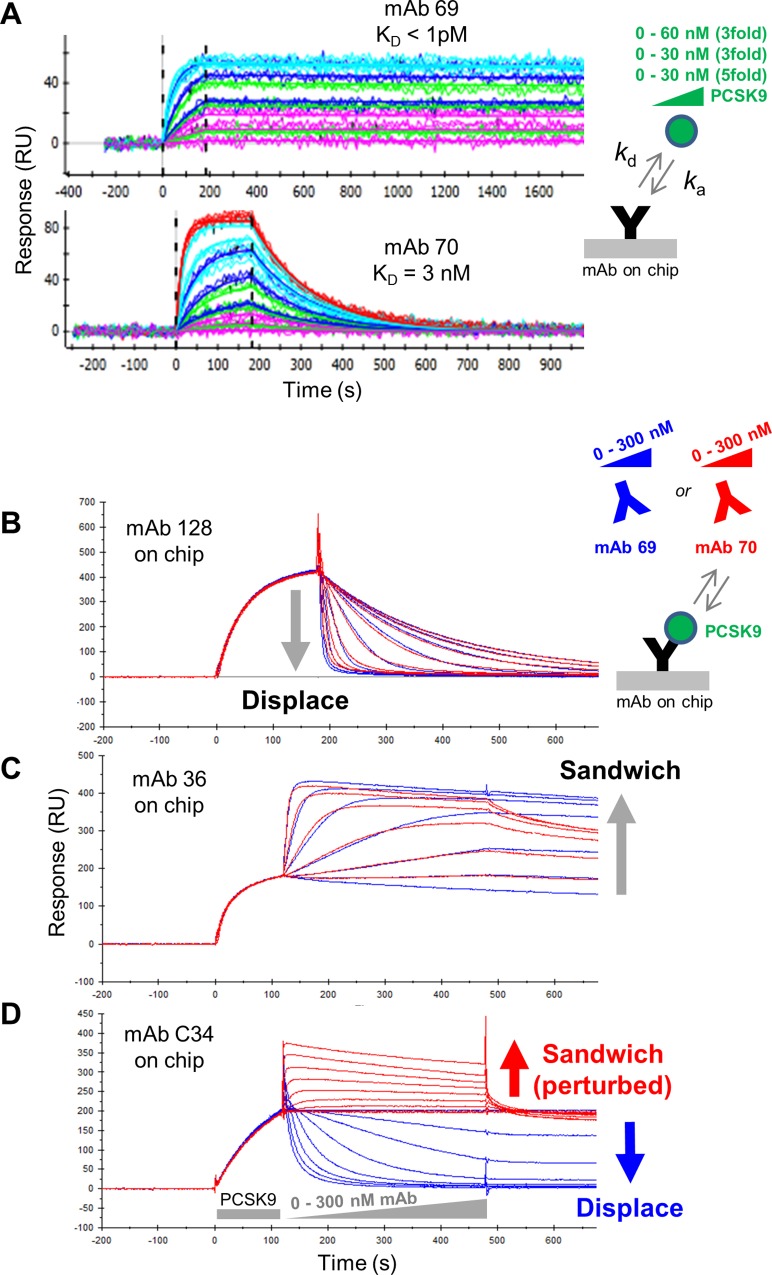
Comparison of two disparate affinity anti-PCSK9 mAbs in their displacement of other mAbs. (A) One-shot kinetic analysis of PCSK9 binding as analyte to mAb 69 and mAb 70 ligands (ProteOn data). Overlay plots of waterfall competition experiments using mAb 69 (blue) or mAb 70 (red) analytes titrated across the same concentration over PCSK9 that is first captured via (B) mAb 128, (C) mAb 36 or (D) mAb C34 ligands (Biacore 2000 data).

We now compare the sandwiching behaviors of mAb 69 and mAb 70 from the above analysis (red cluster, Fig 3C), which cross-block one another but bind PCSK9 with disparate affinities. A one-shot kinetic analysis revealed apparent K_D_ values of < 1 pM for mAb 69 and 3 nM for mAb 70, with their 1,000fold affinity difference driven almost entirely by the difference in their dissociation rate constant (Fig 4A). When mAb 69 and mAb 70 were tested as analyte in a series of concentration-matched waterfall competition experiments against various mAb ligands (whose kinetic rate and affinity constants are provided in Table 1), they were equally potent at displacing mAb 128 ligand (Fig 4B), and sandwich paired in a similar manner with mAb 36 ligand (Fig 4C). Given the disparate affinities of mAb 69 and mAb 70, their superimposable dose-dependent displacement and sandwiching behaviors appeared to be driven by their very similar kinetic association rate constants.

**Table 1:** SPR kinetic and affinity measurements.

MAb	Immobilization	Antigen	k_a_ (M^-1^s^-1^)	k_d_ (s^-1^)	K_D_ (nM)	*N*
C34	Biotin-capture	PCSK9	(0.24–1.2) x 10^6^	(0.75–1.1) x 10^−5^	<0.004	4
69	Biotin-capture	PCSK9	(1.7–3.6) x 10^6^	(0.82–1.5) x 10^−5^	<0.001	4
70	Biotin-capture	PCSK9	2.2 x 10^6^	7.0 x 10^−3^	3	3
128	Biotin-capture	PCSK9	2.7 x 10^5^	3.8 x 10^−3^	14	3
36	Biotin-capture	PCSK9	1.87 x 10^6^	1.80 x 10^−4^	0.10	1
C21	Amine-coupling	PGRN	(2.1, 1.9) x 10^7^	<1.4 x 10^−5^	<0.001	2
28H6 (M27)	Amine-coupling	PGRN	(2.8, 2.4) x 10^6^	(6.3, 4.1) x 10^−5^	0.023, 0.017	2
14C7 (M4)	Amine-coupling	PGRN	(8.3, 8.9) x 10^5^	(5.7, 5.2) x 10^−4^	0.69, 0.58	2
17D7	Amine-coupling	EGFR	(3.7, 5.5) x 10^5^	(1.1, <1.7) x 10^−4^	0.29, <0.31	2
54D7	Amine-coupling	EGFR	(4.4, 6.3) x 10^5^	(0.6, <1.7) x 10^−4^	0.13, <0.27	2
duligotuzumab	Amine-coupling	EGFR	(1.9, 1.8) x 10^6^	(3.5, 3.0) x 10^−3^	1.9, 1.7	2
necitumumab	Amine-coupling	EGFR	(3.9, 2.2) x 10^6^	(5.9, 3.6) x 10^−3^	1.5, 1.6	2
matuzumab	Amine-coupling	EGFR	(3.6, 3.4) x 10^5^	(3.4, 3.1) x 10^−2^	93.1, 92.6	2
cetuximab	Amine-coupling	EGFR	(2.0, 1.7) x 10^6^	(8.5, 8.3) x 10^−4^	0.42, 0.49	2

Data were obtained in a one-shot kinetic method on a ProteOn by injecting monovalent antigens as analytes over mAbs that were either biotinylated and captured onto NLC chips or amine-coupled onto GLC chips. The reported values represent a global analysis of data obtained for *N* independent experiments, except for mAbs 70 and 128 where the data from three independent analyte dilution series were fit simultaneously.

In contrast, mAb C34 ligand could discriminate between mAb 69 and mAb 70 analytes (Fig 4D), since it was displaced by mAb 69 but appeared to sandwich pair with mAb 70. By comparing the shape of the sensorgrams in Fig 4C and 4D, mAb 70’s sandwiching responses are kinetically perturbed towards mAb C34 ligand (Fig 4D) relative to those against mAb 36 ligand (Fig 4C), suggesting that the mAb pair C34/70 cannot co-exist stably on PCSK9. This is confirmed when their competition is studied in the opposite orientation, because mAb C34 analyte displaces mAb 70 ligand (S3 Fig). In summary, Fig 4 (and Table 1) shows that analytes with disparate affinities but similar kinetic association rate constants (mAb 69, K_D_ < 1 pM and mAb 70, K_D_ = 3 nM) can displace lower affinity mAbs (such as mAb 128, K_D_ = 14 nM) with similar potency but may be discriminated in their displacement of higher affinity mAbs (such as mAb C34, K_D_ < 4 pM). This example also illustrates that apparent unidirectional displacement in a heat map resulting in an asymmetry or conflicting blocking assignment for a given mAb pair often occurs between affinity-mismatched pairs, such as C34/70 and C34/3, with displacement being more clearly observed when the higher affinity mAb is used in the role of analyte.

### Antibody displacements occur independent of target and may be missed when competition is tested in a premix assay format

**Fig 5:**
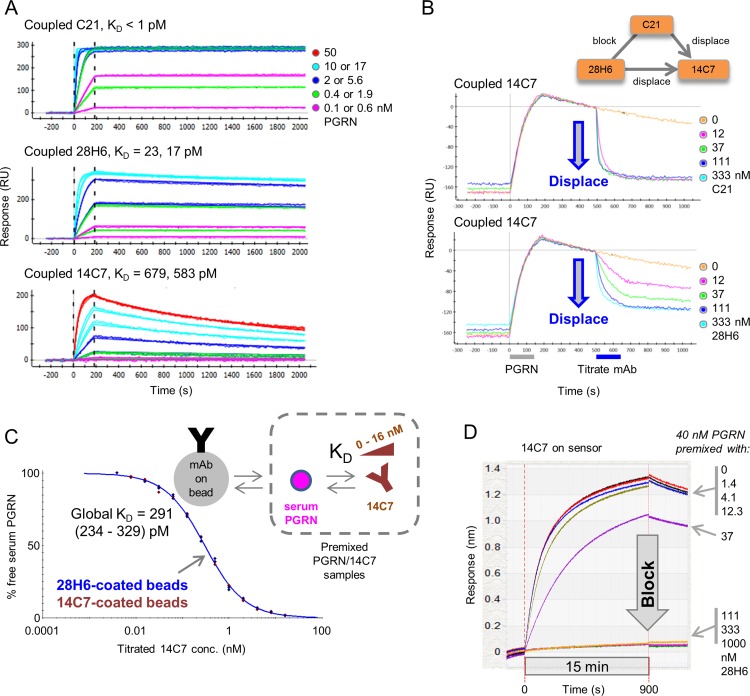
Kinetic and epitope binning characterization of anti-PGRN mAbs that displace one another. (A) One-shot kinetic analysis of PGRN binding to coupled mAbs C21, 28H6 (M27), and 14C7 (M4); see Table 1. (B) Examples of waterfall competition plots for mAb C21 and mAb 28H6 (M27) analytes showing their displacement of mAb 14C7 (M4) ligand (ProteOn data). Blocking relationships for these three mAbs are shown in the inset; C21 and 28H6 block one another and potently displace 14C7, whereas 14C7 blocks (barely displaces) C21 and 28H6. (C) KinExA analysis determining the apparent affinity of 14C7 for serum PGRN as detected using 14C7-coated or 28H6-coated beads. Curves from both bead types are overlaid and fit globally. (D) Premix competition of 14C7 and 28H6 using an Octet-Red384, showing that solution-based 28H6 at concentrations > 40 nM fully blocks binding of 40 nM to 14C7-coated sensors, consistent with an exact titration of binding sites.

We observed several examples of potent mAb displacements when epitope binning a panel of mAbs that target PGRN. To illustrate these displacements, we use mAbs produced from two independent sources, namely mAb C21 generated from the immunization of chickens and mAb 28H6 (also known as M27) and mAb 14C7 (also known as M4) generated from the immunization of mice [15]. A one-shot kinetic analysis of PGRN over immobilized C21, 28H6, and 14C7 yielded apparent K_D_ values of < 2, ~20, and ~ 600 pM (Fig 5A and Table 1). Using a chimeric swap epitope mapping strategy [15], the epitopes of all three mAbs were co-localized to the same subdomain of PGRN, namely granulin E [18], and when tested for cross-blocking in a classical sandwich assay format, C21 and 28H6 blocked one another and both potently displaced 14C7. Upon reversing the assay orientation, 14C7 blocked both C21 and 28H6, and appeared to partially displace them but only when 14C7 was used at high analyte concentrations (1 μM). Consistent with C21 having a higher k_a_ value (and tighter affinity) than 28H6, C21 analyte appeared tenfold more potent than 28H6 analyte at displacing 14C7 (Fig 5B).

We also studied competition of the mAb pair 14C7/28H6 using a premix assay format (Fig 5C). Using the KinExA method, we determined the solution affinity of 14C7 towards native PGRN, as available in human serum. The samples used for these experiments contained a constant concentration of serum antigen that was mixed and equilibrated with titrating levels of 14C7. Probing the free concentration of serum PGRN in these “premixed” samples using 14C7-coated or 28H6-coated beads yielded indistinguishable titration curves, suggesting that 14C7 and 28H6 blocked one another under the kinetically-excluded conditions of a KinExA experiment, which precludes antibody exchange on the bead owing to the brief contact time used.^3^ When a set of equilibrated premixed samples (containing 40 nM purified recombinant PGRN titrated with 0–1000 nM 28H6 binding sites) was analyzed on the Octet, allowing longer contact time with the solid phase (in this case, immobilized 14C7), a clear blocking result was obtained in both assay orientations (Fig 5D), consistent with an exact titration of binding sites. Taken together, these observations demonstrate that displacements may be missed when competition between disparate affinity mAbs is examined in a premix assay format.

### Structural data imply that antibodies targeting closely adjacent or minimally overlapping epitopes can displace one another

**Fig 6:**
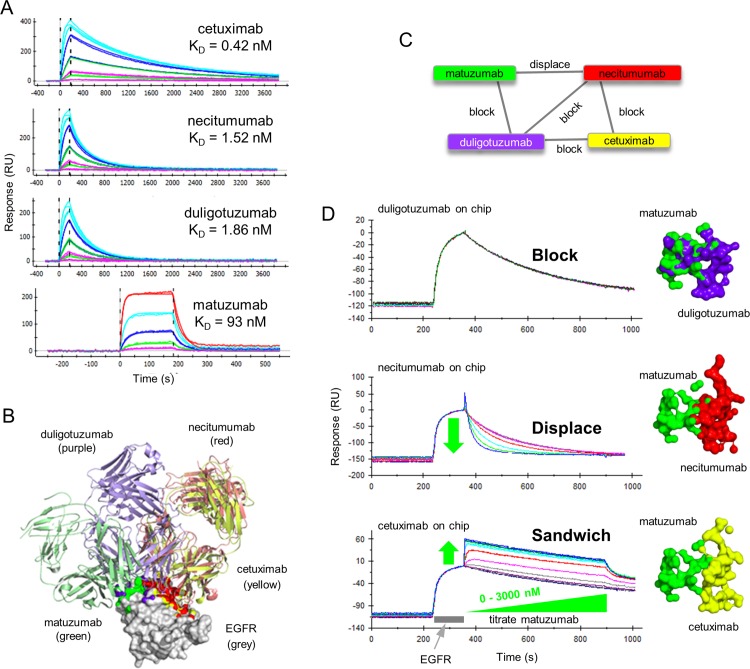
Kinetic and epitope characterization of four literature anti-EGFR mAbs. (A) One-shot kinetic analysis of EGFR binding to amine-coupled mAbs. EGFR was injected over cetuximab, necitumumab, and duligotuzumab as both a 5fold and 3fold dilution series with top at 56 nM. EGFR was injected over matuzumab as a 3fold series with top at 278 nM. (B) Image of Fab:EGFR complexes when superposed via their EGFR domain III. EGFR domain III is shown in surface representation (white) while the different Fab fragments are shown in ribbon representation. Atoms that constitute the epitope for each Fab are colored in yellow (cetuximab), green (matuzumab), purple (duligotuzumab), and red (necitumumab). Buried surface areas were calculated as described under Materials and Methods. (C) Network blocking plot summarizing the empiric cross-blocking results. (D) Left panel—Waterfall competition plots obtained on a Biacore 2000 showing the empiric outcomes of block, displace, or sandwich, when matuzumab analyte is paired with duligotuzumab, necitumumab, or cetuximab ligands, respectively. Right panel—Surface representation of EGFR atoms that are buried upon complex formation for each of the mAbs.

We employed epidermal growth factor receptor (EGFR) as a third model antigen to explore the displacement phenomenon further because, not only had we identified a subset of anti-EGFR mAbs that potently displaced one another, but we could interpret our empiric cross-blocking experiments structurally by including several anti-EGFR mAbs from the literature where structures of the mAb:EGFR complex were available [3–6]. A one-shot kinetic analysis of EGFR binding to four literature mAbs showed that their apparent affinities ranked as follows; cetuximab > necitumumab = duligotuzumab > matuzumab (Fig 6A). Superposition of EGFR from the four mAbs:EGFR DIII complex structures shows that necitumumab (red) and cetuximab (yellow) share very similar epitope footprints (Fig 6B), yet our cross-blocking experiments were able to resolve fine differences between them (Fig 6C). While the four mAbs were able to cross-block at least one other mAb in the test set, each mAb belonged to its own epitope bin because, (a) duligotuzumab blocked all mAbs, (b) cetuximab blocked duligotuzumab and necitumumab but not matuzumab, (c) necitumumab blocked duligotuzumab and cetuximab and displaced matuzumab, and (d) matuzumab blocked duligotuzumab, displaced necitumumab, and did not block cetuximab (Figs 6D and S4). A structural interpretation of these empiric cross-blocking results suggests that displacement occurs between mAbs that have minimal to no steric clashes or shared epitope contacts, reinforcing our proposed hypothesis of displacement occurring via a transient sandwich complex within which the mAbs kinetically perturb one another, resulting in the complex collapsing by expelling one mAb and retaining the other.

**Fig 7:**
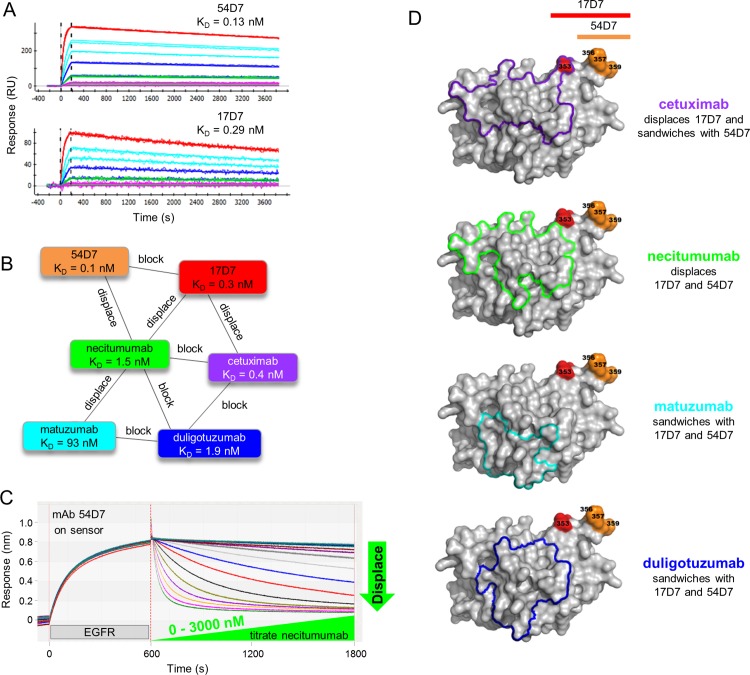
Kinetic and epitope characterization of in-house anti-EGFR mAbs. (A) One-shot kinetic analysis of EGFR analyte (injected as both a 5fold and 3fold dilution series with top at 56 nM) binding to coupled mAbs 54D7 and 17D7. (B) Network blocking plot summarizing the empiric cross-blocking results for a panel of six anti-EGFR mAbs. Chords between two mAbs indicate a reversible blocking pair or a reversible displacement, as indicated. No chord between two mAbs indicates a sandwiching pair, e.g., 54D7/duligotuzumab. (C) Waterfall competition plot obtained on an Octet-Red384 showing that necitumumab analyte (as a 14-membered twofold series with top at 6200 nM) dose-dependently displaces 54D7 ligand. (D) Surface representation of EGFR (shown in grey) with an outline of residues that are buried upon complex formation with each mAb. This representation differs from that shown in Fig 6 in that the outline comprises all residues that have change in surface burial, as opposed to showing only individual atoms that change burial. Thus Fig 6 is a higher resolution representation of the buried epitope and Fig 7D can be considered a lower resolution epitope representation. The residues identified from mutagenesis-based epitope mapping for mAb 17D7 (red) and mAb 54D7 (orange) are shown. Residues buried upon each Fab binding are shown in as an outline. Buried surface areas were calculated as described under Materials and Methods.

A one-shot kinetic analysis of our in-house anti-EGFR mAbs 54D7 and 17D7 showed that they bound EGFR stably and with high affinity (Fig 7A and Table 1). They were chosen for this study because they block one another and displace some of the literature mAbs; 54D7 and 17D7 displace necitumumab, but only 17D7 displaces cetuximab (Fig 7B). To illustrate that disparate affinity mAbs can reversibly displace one another, in Fig 7C we show that necitumumab analyte (K_D_ = 1.5 nM) can potently displace 54D7 ligand (K_D_ = 0.13 nM). Indeed, when a waterfall competition analysis was performed in opposing assay orientations for 54D7 and necitumumab, they displaced one another with similar potency despite their tenfold affinity difference (S5 Fig); this example highlights that the kinetic association rate constants (k_a_ values) of the antigen/mAb binding interactions involved can influence the potency of displacements, because 54D7’s k_a_ value is around tenfold smaller than necitumumab’s, thereby offsetting their affinity difference (Table 1).

In the absence of structural data for mAbs 54D7 and 17D7, we epitope mapped them using a high-throughput site-directed antigen mutagenesis strategy (see Materials and Methods) to obtain structural insight for their displacement behaviors. We prepared a set of EGFR mutants and screened their binding to 54D7, 17D7, and cetuximab (as a control) using the Octet to identify critical binding residues. The epitope mapping results revealed that 54D7’s functional epitope (S356, F357, and H359) was similar to 17D7’s functional epitope (R353, S356, F357, and H359) and did not overlap with cetuximab’s functional epitope (H409, K465, and I467). The single residue in 17D7’s functional epitope (R353) that was absent from 54D7’s functional epitope was located at the periphery of cetuximab’s structural epitope footprint (Fig 7D). Indeed, necitumumab’s structural epitope footprint was also at the periphery of R353 and includes residues that are closer to 54D7’s functional epitope when compared to cetuximab’s structural epitope footprint.

**Fig 8:**
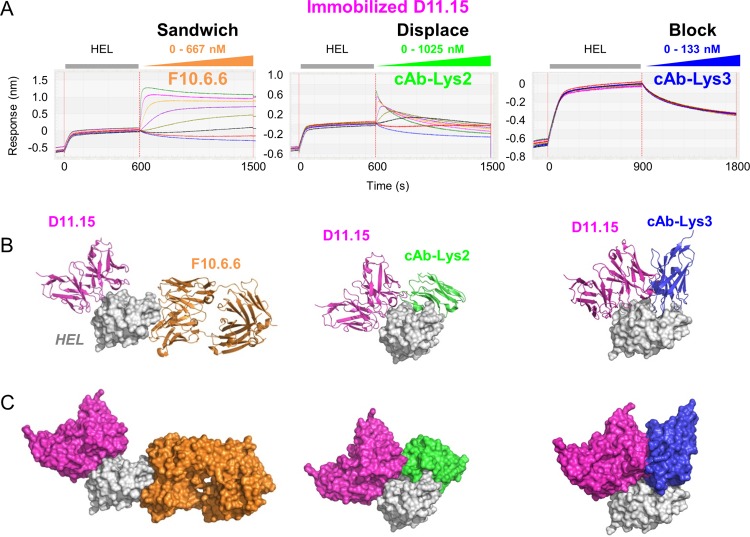
Cross-blocking analysis of literature anti-HEL mAbs. (A) Waterfall competition plot obtained on an Octet-Red384 showing outcomes of sandwich, displacement (kinetically perturbed sandwiching), and block for the titrated mAb analytes F10.6.6, cAb-Lys2, and cAb-Lys3 when tested for binding to HEL that is captured via biotinylated mAb D11.15 (ligand) coated onto streptavidin sensors. Each mAb analyte was titrated as a 3fold serial dilution at the concentration range indicated (in binding sites). (B) Ribbon representations of the structures of D11.15 (pink), F10.6.6 (orange), cAb-Lys2 (green), and cAb-Lys3 (blue) when superposed on HEL (light grey surface representation), showing that D11.15 and F10.6.6 bind at spatially separated epitopes, whereas D11.15 has some steric clashes with cAb-Lys2 and shares minimal epitope contacts with cAb-Lys3. (C) Surface representation of the structures shown in panel B.

To provide additional examples and gain further structural insight into the mechanism of antibody displacement on an independent model system, we selected a small panel of mAbs from the literature with crystallographically-defined epitopes on hen egg lysozyme (HEL)[7–10]. Fig 8A shows that waterfall competition experiments on immobilized mAb D11.15, using F10.6.6, cAb-Lys2, and cAb-Lys3 mAb analytes, yielded different sandwiching profiles; D11.15 clearly sandwich paired with F10.6.6, D11.15 formed a transient sandwich complex with cAb-Lys2, and D11.15 clearly blocked cAb-Lys3. The images in Fig 8B represent the superposed co-crystal complexes of the above-mentioned mAb pairs, providing a structural interpretation of their empiric cross-blocking results. The clear sandwiching of D11.15 with F10.6.6 is expected due to their well-separated and non-overlapping epitopes. Based on the crystal structures, one might expect cross-blocking results for both D11.15/cAb-Lys3 and D11.15/cAb-Lys2, however, cross-blocking is observed only for the D11.15/cAb-Lys3 pair. To rationalize the transient sandwiching complex observed for the mAb pair D11.15/cAb-Lys2, we speculate a potential rearrangement of one loop on cAb-Lys2, whereas the larger and more rigid overlap of the epitopes of the mAb pair D11.15/cAb-Lys3 could not accommodate a trimolecular complex and so a block is observed. This example highlights that an interplay of subtle differences in epitope contacts, steric clashes, and the potential for rearrangement of antibody loops influence whether two mAbs cross-block or displace one another. It also suggests that displacement of one antibody by another can occur even if the antibody epitopes overlap to some small extent.

## Discussion

By leveraging epitope binning data of mAbs with crystallographically-defined epitopes for model antigens EGFR and HEL, we have gained insight into the structural requirements for displacements. In the examples studied herein, mAbs with greatly overlapping epitopes appeared to block one another, whereas those with closely adjacent or minimally overlapping epitopes appeared to displace one another. Whether displacement can occur between mAbs with significantly overlapping epitopes would necessitate further investigation using the approach described herein, namely the use of label-free methods to identify antibodies that displace one another and corroboration with structural data defining their epitopes, as available from crystallographic studies or from epitope mapping studies that utilize site-directed antigen mutagenesis. Antibody displacements manifesting due to mAbs profoundly altering one another’s binding kinetics towards their specific antigen when both mAbs co-exist momentarily on their antigen in the form of a transient sandwich complex, can serve to further refine epitope binning assignments and provide additional metrics for their differentiation. Displacing mAbs might be utilized for gentle, neutral pH elution in protein purification and might also have novel therapeutic applications. For example, if the epitope of an anti-receptor mAb is closely adjacent to the receptor’s natural binding partner (its cognate ligand), ligand displacement may provide a more potent mechanism of ligand competition than the traditional ligand blockade offered by a mAb that shares a substantial part of the ligand’s epitope.

Our study shows that, when epitope binning experiments are performed on label-free biosensors in a classical sandwich assay format an analyte can exhibit a range of sandwiching responses with a ligand due to an interplay of both, the relative binding kinetics of the analyte and ligand towards their specific antigen and the proximity of their epitopes to one another. In the most extreme case, an analyte can fully displace a ligand, as judged by analytes that cause a significant loss of signal upon their binding resulting in an inverted sandwiching signal that drops to a baseline response, representing the naked ligand, as shown in Fig 2C, where the analyte (mAb 69) fully displaces the antigen (PCSK9) from the ligand (mAb C34). However, not all examples of displacement result in the antigen departing with the analyte. For example, in Fig 8A (middle panel), the analyte (cAb-Lys2) forms an initial sandwiching complex, which decays during the sandwiching step, resulting in the antigen (HEL) remaining bound to the ligand (D11.15). This behavior is also observed in Fig 4D, where even though the C34/PCSK9/70 sandwich complex is unstable, it persists during the sandwiching step, but rapidly collapses during the buffer dissociation step leaving the antigen bound to the ligand (C34). In S6 Fig, we provide additional examples of antibody pairs (anti-PCSK9 mAbs 131/28, 131/36, and 63/36) that appear to displace one another via the formation of a transient sandwiching complex that collapses by expelling the analyte, leaving the antigen bound to the ligand.Our data show that the potency of a mAb displacement is not only influenced by the proximity of the epitopes targeted by the competing mAbs in question, but by their relative affinities and importantly, their k_a_ values towards their specific antigen. Our waterfall competition plots illustrate the dose-dependent nature of displacements, showing a spectrum of perturbed sandwiching profiles when competition is examined across a range of analyte concentrations. In contrast, when competition is examined at a single analyte concentration, as routinely employed in high throughput screening mode, some mAb pairs may be misclassified as giving discordant blocking assignments, confounding an interpretation of an epitope binning experiment. Even though mAb displacement is fully reversible in principle, displacement may be more easily observed in one assay orientation than another if the competing mAbs have disparate affinities (or disparate k_a_ values); in this case, the mAb with the tighter affinity (or faster k_a_) will appear to displace the other more potently when displacements are examined at the same analyte concentration.In addition to apparent cases of asymmetric blocking in a binning experiment, two mAbs that kinetically perturb one another’s binding to their specific antigen may manifest as unusually small sandwiching responses that are assigned to the “ambiguous/intermediate” category because the mAbs neither block nor sandwich one another clearly. This highlights an opportunity for software that can identify these behaviors and facilitate the identification of mAb pairs that might require follow up studies like waterfall competition that could be performed using the same assay configuration. For example, a simple multi-report point analysis could be employed, that would flag analytes that displace ligands and monitor the initial transient sandwiching complex, if detectable, and recommend them for further study using the same surfaces. Thus, the ability of real-time label-free biosensors to monitor kinetic perturbations occurring within transient sandwich complexes enables the identification of antibody displacements that would be overlooked by most other analytical methods. The short-range effect of the displacement phenomenon is distinct from the traditional long-range effect reported for allosteric interactions, and could be employed in a variety of reagent or therapeutic settings where nuanced specificity is desired.

## Supporting Information

S1 Fig Premix blocking results showing the competition of anti-PCSK9 mAbs 69 and C34. Binding responses obtained for 50 nM PCSK9 premixed and equilibrated with 0, 5.6, 16.7, 50, 150, 450, or 1350 nM binding sites of mAb 69 (A and B) or mAb C34 (C and D), when bound to mAb 69-coated sensors (A and C) or mAb C34-coated sensors (B and D). Data were obtained on the Octet-Red-384 equipped with streptavidin sensors. A full block consistent with an exact titration of 50 nM binding sites was obtained in panels A and D, whereas a partial block was observed in panels B and C, suggesting that competition of mAb69 with mAb C34 involved detection of free PCSK9 and a transient sandwich complex of 69/PCSK9/C34.
(TIF)

S2 Fig SPR imaging data showing examples of order-dependent blocking for the mAb pair 63/24. Top panel shows the overlay plots for a panel of 43 mAb analytes binding to PCSK9 that is first captured via (A) mAb 63 ligand or (B) mAb 24 ligand. Bottom panel shows select curves relative to the buffer blank curves (shown in blue); (C) mAb 24 analyte is blocked when injected over mAb 63 ligand whereas (D) mAb 63 analyte sandwich pairs with mAb 24 ligand.
(TIF)

S3 Fig SPR imaging data showing examples of order-dependent blocking (or displacement), manifesting as apparent asymmetries in the binning heat map. (A) Overlay plot showing various analytes (mAbs 3, 70, 61, 25, 69, and C34) binding to PCSK9 that is first captured via C34 ligand and (B) the same mAb pairings analyzed in the opposite orientation, with C34 as analyte. (C) Summary of the binning outcomes, representing a subset of the data shown in Fig 3D.
(TIF)

S4 Fig Surface representation of Fab fragments of various antibodies and their EGFR epitopes. The epitopes are represented as in Fig 6D but rotated by 90 degrees. (A) Matuzumab (green) and duligotuzumab (blue) have significant overlap of their Fab domains. Consequently, these two antibodies block each other. (B) Matuzumab (green) and necitumumab (red) Fabs bind closely adjacent epitopes on EGFR with potentially minor overlap in the Fab region. Such interactions resulted in their displacement. (C) Matuzumab (green) and cetuximab (yellow) Fabs bind close to one other, but with no overlap. These two mAbs empirically sandwich with each other.
(TIF)

S5 Fig Examples of antibody pairs (anti-PCSK9 mAbs 131/28, 131/36, and 63/36) that show transient sandwiching, resulting in the displacement of the incoming analyte, rather than the ligand. Panels A and B show the sandwiching results from opposing assay orientations. When assigning blocking or sandwiching classifications to antibody pairs using an end-point analysis, the response is recorded at the end of the analyte injection, which is ~500 sec in these examples, as indicated by the black vertical line. By an end-point analysis, 131/28 and 131/36 appeared blocked in both orientations, whereas 63/36 appeared blocked in only one orientation (when mAb 36 is the analyte). However, inspection of their real-time sandwiching profiles reveals that all pairs undergo some degree of displacement. Data were obtained on an SPR imager.
(TIF)

S6 Fig Characterization of disparate affinity anti-EGFR mAbs, 54D7 and necitumumab. (A) One-shot kinetic analysis of EGFR binding as analyte to amine-coupled ligands, 54D7 (top panel) or necitumumab (bottom panel). (B) Waterfall competition plots showing the dose-dependent displacement of 54D7 ligand by necitumumab analyte (top panel) or necitumumab ligand by 54D7 analyte (bottom panel). Data were obtained on a ProteOn XPR36 biosensor.
(TIF)
