# Supplementary figures and images for: Antibodies Targeting Closely Adjacent or Minimally Overlapping Epitopes Can Displace One Another
Source: PLoS One. 2017 Jan 6;12(1):e0169535. doi: 10.1371/journal.pone.0169535 (PMC5218414; doi:10.1371/journal.pone.0169535)

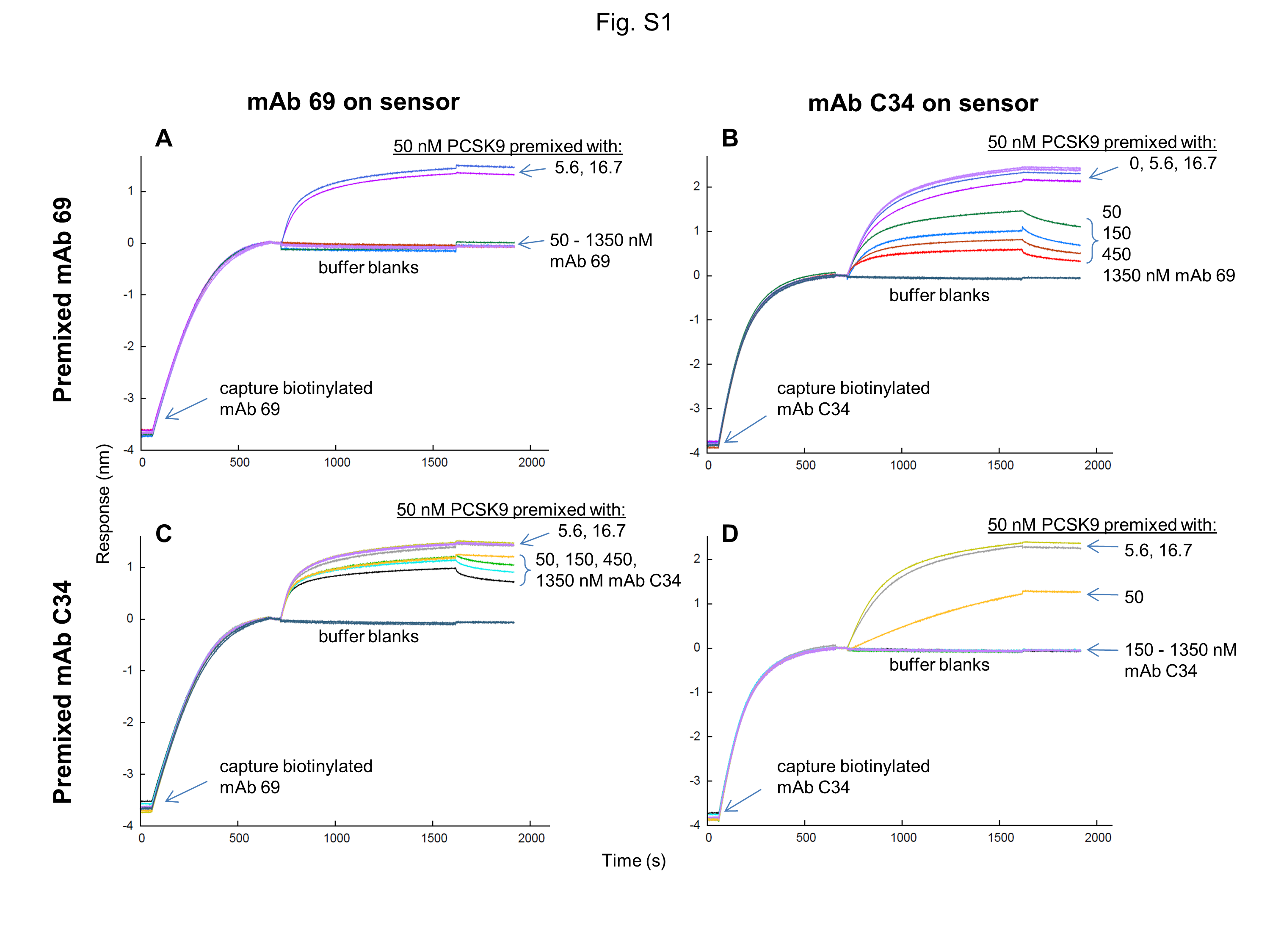

Supplement: S1 Fig — Binding responses obtained for 50 nM PCSK9 premixed and equilibrated with 0, 5.6, 16.7, 50, 150, 450, or 1350 nM binding sites of mAb 69 (A and B) or mAb C34 (C and D), when bound to mAb 69-coated sensors (A and C) or mAb C34-coated sensors (B and D). Data were obtained on the Octet-Red-384 equipped with streptavidin sensors. A full block consistent with an exact titration of 50 nM binding sites was obtained in panels A and D, whereas a partial block was observed in panels B and C, suggesting that competition of mAb69 with mAb C34 involved detection of free PCSK9 and a transient sandwich complex of 69/PCSK9/C34. (TIF) [file pone.0169535.s001.TIF]

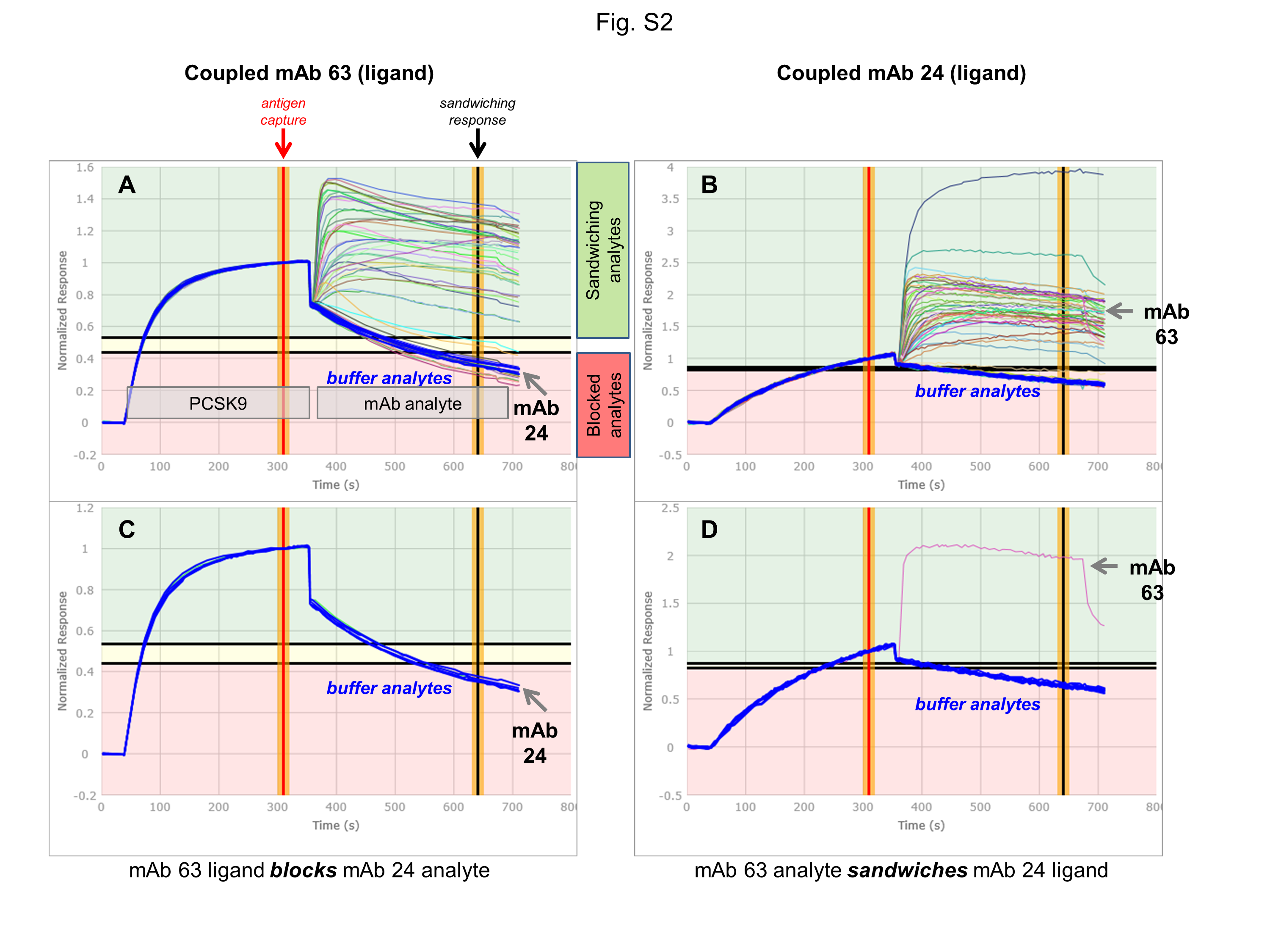

Supplement: S2 Fig — Top panel shows the overlay plots for a panel of 43 mAb analytes binding to PCSK9 that is first captured via (A) mAb 63 ligand or (B) mAb 24 ligand. Bottom panel shows select curves relative to the buffer blank curves (shown in blue); (C) mAb 24 analyte is blocked when injected over mAb 63 ligand whereas (D) mAb 63 analyte sandwich pairs with mAb 24 ligand. (TIF) [file pone.0169535.s002.TIF]

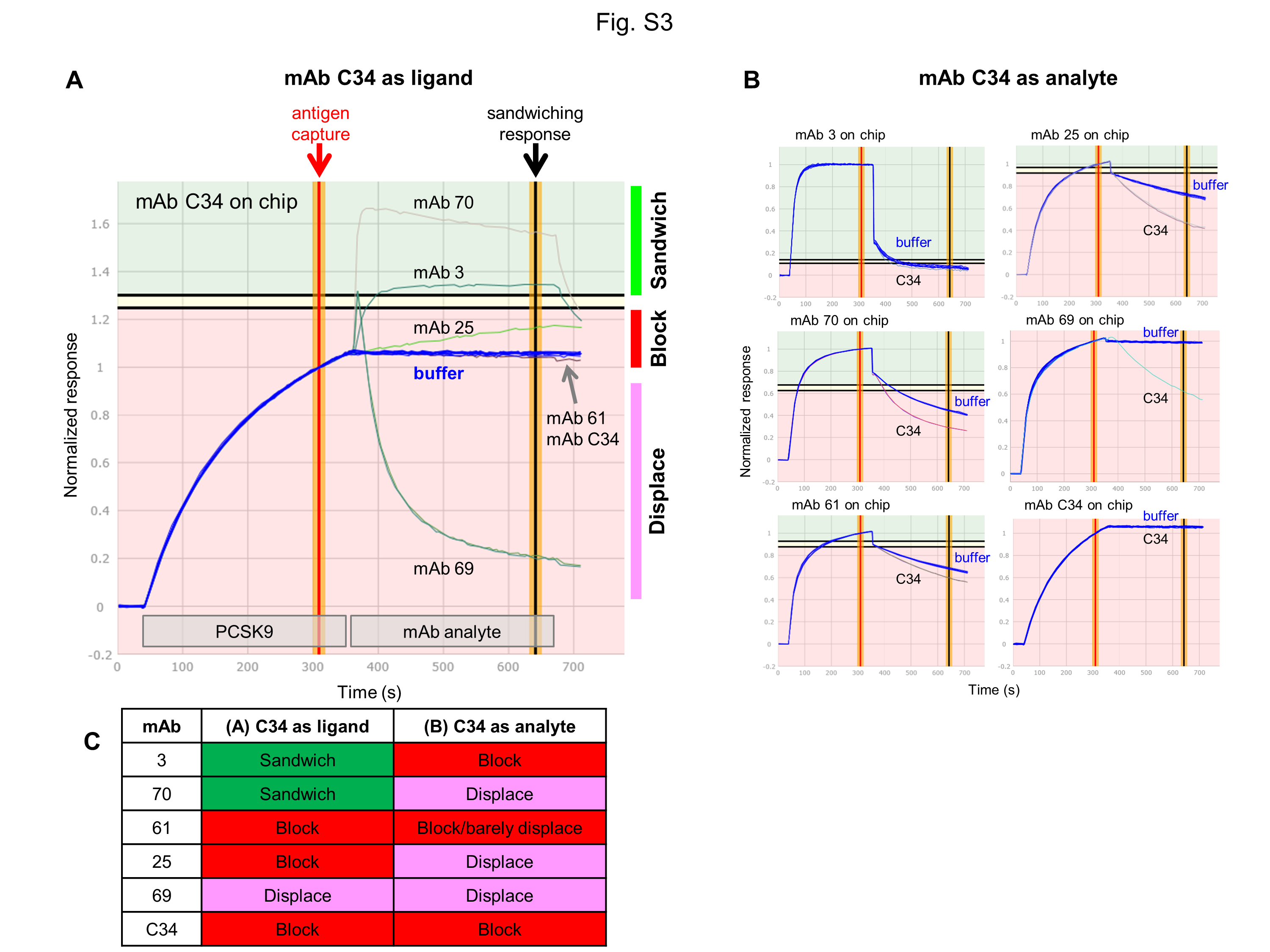

Supplement: S3 Fig — (A) Overlay plot showing various analytes (mAbs 3, 70, 61, 25, 69, and C34) binding to PCSK9 that is first captured via C34 ligand and (B) the same mAb pairings analyzed in the opposite orientation, with C34 as analyte. (C) Summary of the binning outcomes, representing a subset of the data shown in Fig 3D. (TIF) [file pone.0169535.s003.TIF]

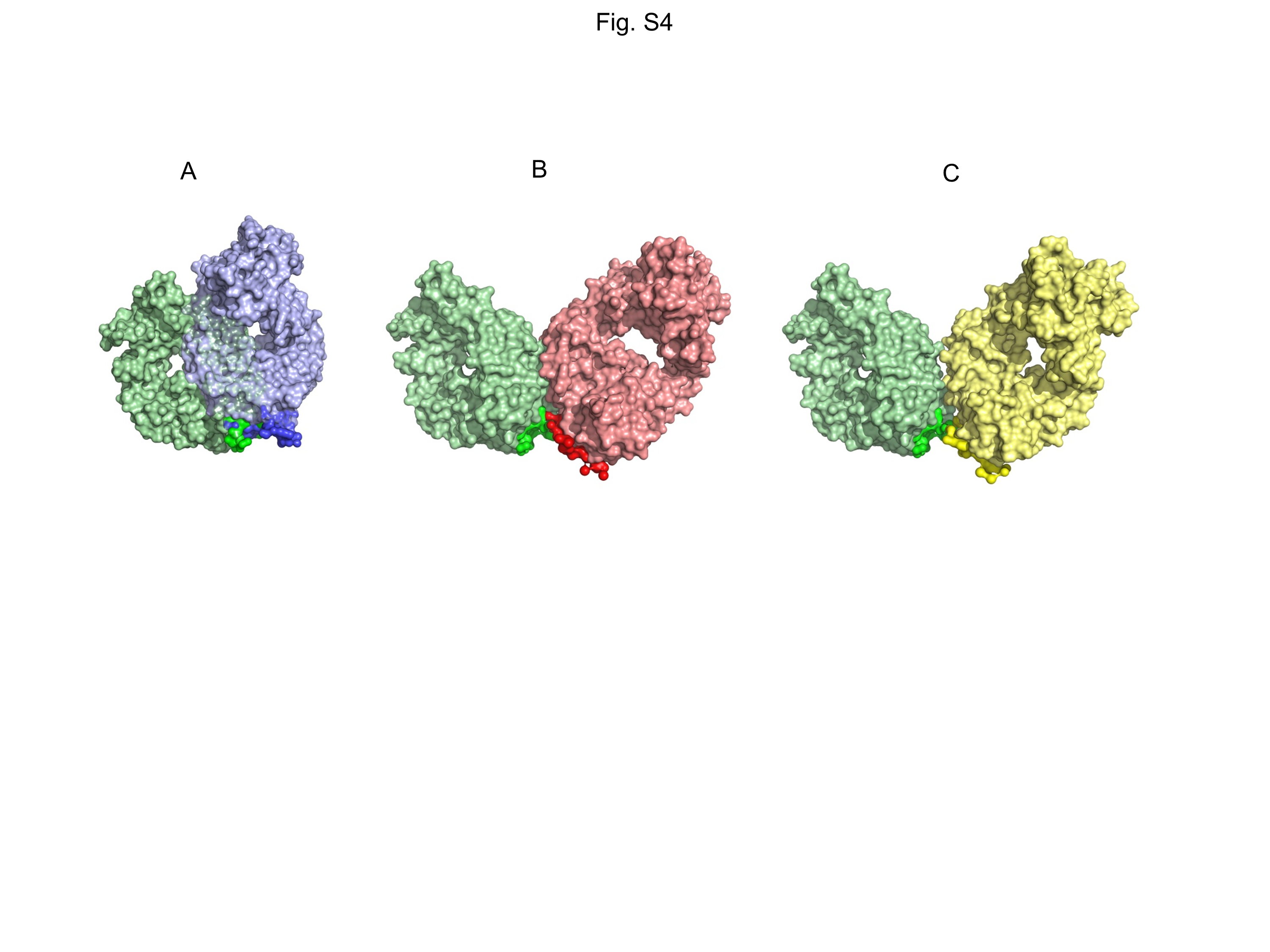

Supplement: S4 Fig — The epitopes are represented as in Fig 6D but rotated by 90 degrees. (A) Matuzumab (green) and duligotuzumab (blue) have significant overlap of their Fab domains. Consequently, these two antibodies block each other. (B) Matuzumab (green) and necitumumab (red) Fabs bind closely adjacent epitopes on EGFR with potentially minor overlap in the Fab region. Such interactions resulted in their displacement. (C) Matuzumab (green) and cetuximab (yellow) Fabs bind close to one other, but with no overlap. These two mAbs empirically sandwich with each other. (TIF) [file pone.0169535.s004.TIF]

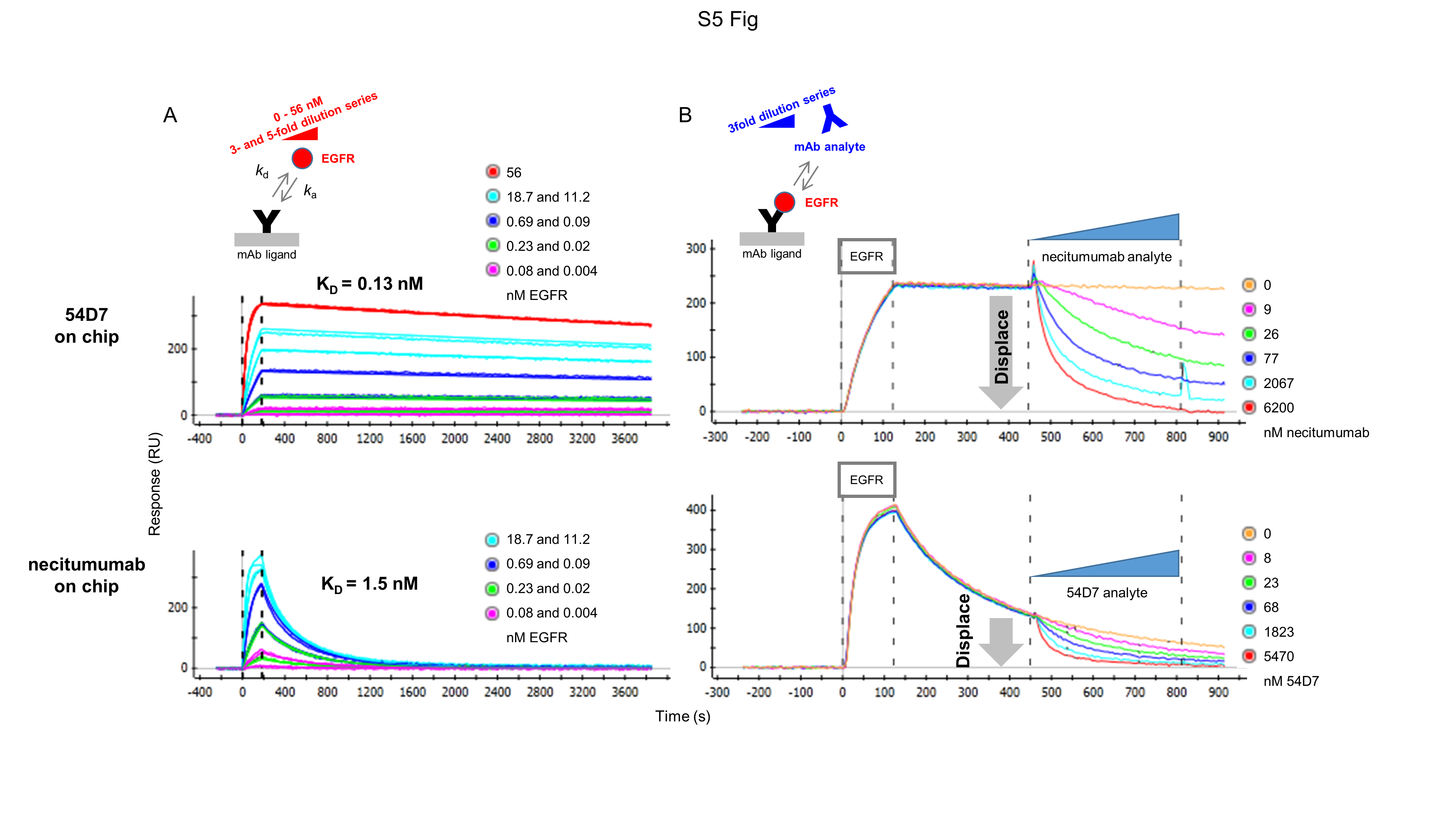

Supplement: S5 Fig — Panels A and B show the sandwiching results from opposing assay orientations. When assigning blocking or sandwiching classifications to antibody pairs using an end-point analysis, the response is recorded at the end of the analyte injection, which is ~500 sec in these examples, as indicated by the black vertical line. By an end-point analysis, 131/28 and 131/36 appeared blocked in both orientations, whereas 63/36 appeared blocked in only one orientation (when mAb 36 is the analyte). However, inspection of their real-time sandwiching profiles reveals that all pairs undergo some degree of displacement. Data were obtained on an SPR imager. (TIF) [file pone.0169535.s005.TIF]

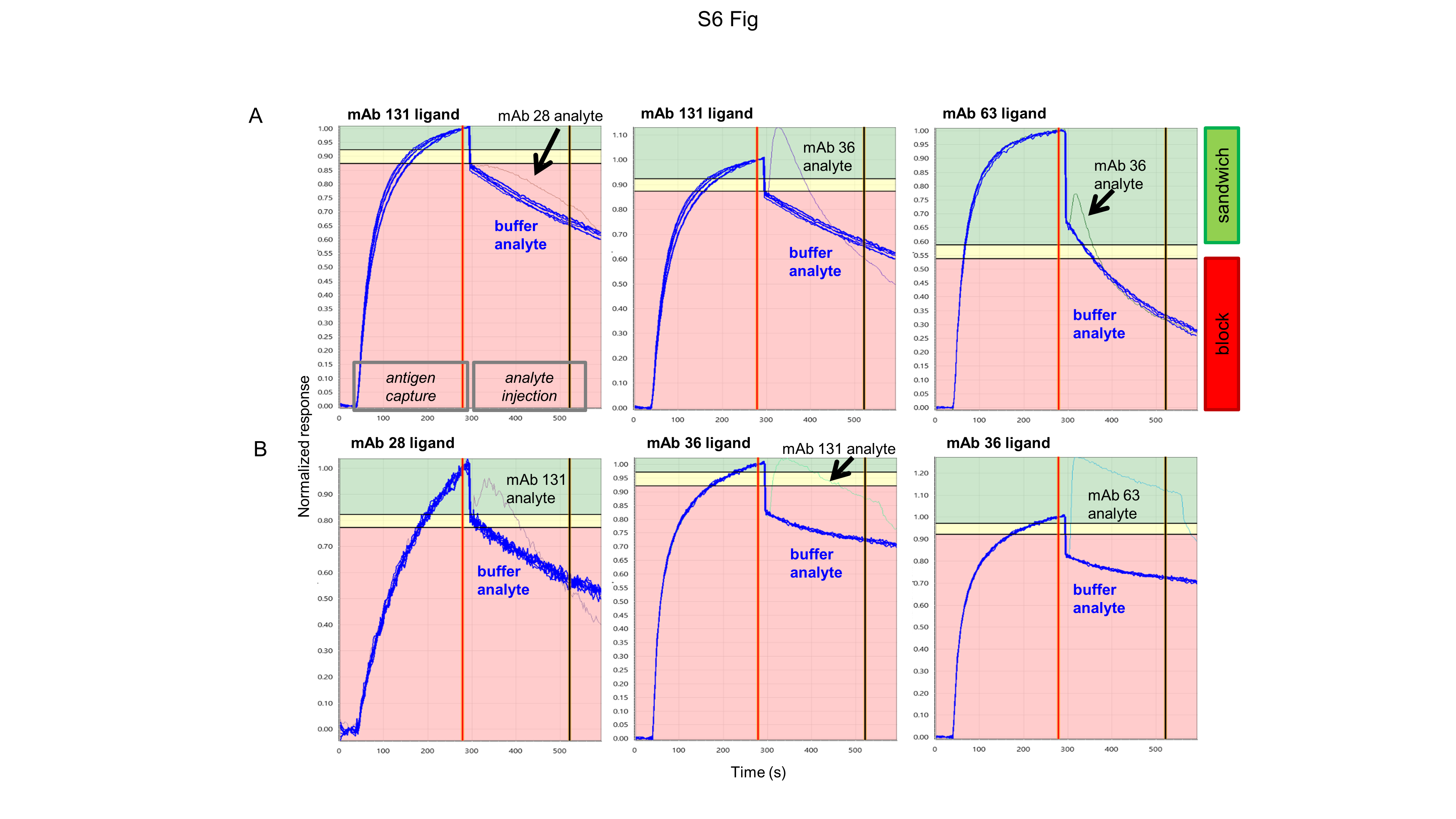

Supplement: S6 Fig — (A) One-shot kinetic analysis of EGFR binding as analyte to amine-coupled ligands, 54D7 (top panel) or necitumumab (bottom panel). (B) Waterfall competition plots showing the dose-dependent displacement of 54D7 ligand by necitumumab analyte (top panel) or necitumumab ligand by 54D7 analyte (bottom panel). Data were obtained on a ProteOn XPR36 biosensor. (TIF) [file pone.0169535.s006.TIF]
